# Perivascular diffusion and white matter free water improve identification of cognitive impairment in cerebral small vessel disease

**DOI:** 10.3389/fneur.2026.1879894

**Published:** 2026-08-03

**Authors:** Chunhong Yang, Sunmei Cai, Bing Wang, Jie Zhou, Dongmei Wu, Muzi Li, Wenlu Geng, Lianshuang Wu, Kaiyue Peng, Yi Yang, Wenquan Gu

**Affiliations:** 1 Department of Radiology, Punan Branch of Renji Hospital, Shanghai Jiaotong University School of Medicine (Punan Hospital in Pudong New District, Shanghai), Shanghai, China; 2 Shanghai Key Laboratory of Magnetic Resonance, School of Physics, Institute of Magnetic Resonance and Molecular Imaging in Medicine, East China Normal University, Shanghai, China; 3 School of Engineering and Applied Science, University of Pennsylvania, Philadelphia, PA, United States; 4 Department of Neurology, Punan Branch of Renji Hospital, Shanghai Jiaotong University School of Medicine (Punan Hospital in Pudong New District, Shanghai), Shanghai, China; 5 Department of Nuclear Medicine, The First Affiliated Hospital of Soochow University, Suzhou, China

**Keywords:** cerebral small vessel disease, cognitive impairment, diffusion tensor imaging, free water, glymphatic system, neuroimaging biomarkers

## Abstract

**Inroduction:**

Cerebral small vessel disease contributes substantially to cognitive decline in older adults, but conventional structural magnetic resonance imaging markers mainly reflect accumulated lesion burden and do not directly characterize diffusion-derived alterations in brain fluid homeostasis. We investigated whether combining diffusion imaging along the perivascular space and white matter free-water imaging improves identification of Montreal Cognitive Assessment (MoCA)-defined cognitive impairment in elderly patients with cerebral small vessel disease.

**Methods:**

This prospective study included 100 patients with cerebral small vessel disease and 50 healthy controls. According to MoCA scores, patients were classified as cognitively normal (*n* = 42) or cognitively impaired (*n* = 58). All participants underwent 3.0-T magnetic resonance imaging, including conventional structural imaging and diffusion tensor imaging. Conventional structural magnetic resonance imaging markers were evaluated on anatomical imaging, whereas diffusion-derived metrics were used to assess perivascular diffusion and free-water burden. Group comparisons, correlation analyses, multivariate logistic regression, and receiver operating characteristic analyses were performed.

**Results:**

Analysis along the perivascular space decreased progressively from healthy controls to cognitively normal patients and cognitively impaired patients, whereas white matter free water increased in the opposite direction (all *p* < 0.001). Lower ALPS-Index values and higher white matter free-water values were both associated with lower MoCA scores. The clinical information model showed moderate discrimination for MoCA-defined cognitive impairment (area under the curve = 0.764, 95% confidence interval: 0.669–0.859). After incorporating age, sex, education, hypertension, and diabetes as covariates, the traditional cerebral small vessel disease marker model achieved an area under the curve of 0.819 (95% confidence interval: 0.731–0.908), whereas the glymphatic-edema signature combining ALPS-Index and white matter free water achieved better discriminative performance (area under the curve = 0.928, 95% confidence interval: 0.875–0.981). The combined imaging model incorporating conventional structural markers, ALPS-Index, and white matter free water achieved the highest discriminative performance (area under the curve = 0.950, 95% confidence interval: 0.906–0.993).

**Discussion:**

Perivascular diffusion imaging and white matter free water capture complementary aspects of altered perivascular diffusion and extracellular free-water accumulation in cerebral small vessel disease and improve identification of MoCA-defined cognitive impairment beyond conventional structural magnetic resonance imaging markers.

## Introduction

Cerebral small vessel disease (CSVD) represents a major global health challenge, driving approximately 25% of all ischemic strokes and contributing to nearly 45% of dementia cases (1). Traditional magnetic resonance imaging (MRI) markers—including white matter hyperintensities (WMH), lacunar infarcts, and enlarged perivascular spaces (EPVS)—enable clinicians to quantify the structural burden of vascular brain injury (2). Traditional MRI markers such as WMH, lacunes, and EPVS primarily reflect accumulated structural burden rather than directly measuring ongoing disturbances in brain fluid transport and tissue homeostasis (2, 3). Nevertheless, these structural markers should not be regarded as strictly irreversible end-stage lesions, because longitudinal studies have reported regression or dynamic change in WMH and focal perivascular spaces (4, 5). As the elderly population expands, neuroimaging must transition from merely describing established lesions to probing upstream mechanisms, particularly altered perivascular diffusion (6).

The glymphatic system functions as a microscopic waste-clearance network, facilitating the exchange of cerebrospinal fluid (CSF) and interstitial fluid (ISF) along perivascular channels (7). In the aging brain, arterial stiffening and venous congestion disrupt this delicate fluid transport, leading to metabolic waste accumulation and subsequent axonal injury (8). Diffusion tensor imaging analysis along the perivascular space (DTI-ALPS) offers a powerful, contrast-free diffusion-derived surrogate of perivascular diffusion (9). By measuring water diffusivity parallel to the deep medullary veins, the ALPS-Index provides a diffusion-derived surrogate of perivascular fluid transport, which frequently declines in individuals with a high burden of CSVD (10). Recent CSVD studies have further linked reduced ALPS-Index to greater perivascular space burden, hypertension-related CSVD changes, and poorer cognitive performance (11, 12).

Parallel to clearance failure, blood–brain barrier (BBB) dysfunction and other tissue-level processes may contribute to extracellular fluid accumulation and chronic interstitial edema (13). Traditional diffusion metrics often lack the specificity to isolate this fluid component, as they mix signals from cellular tissue and surrounding fluid (14). Advanced bi-tensor modeling, specifically free-water elimination diffusion tensor imaging (FW-DTI), addresses this limitation by partitioning the diffusion signal (14). This approach allows quantification of the FW fraction, an imaging marker for extracellular FW accumulation/interstitial edema that often increases in the “penumbra” of normal-appearing tissue before it converts into visible WMH (15, 16).

Although DTI-ALPS and white matter free water (WM-FW) have each been investigated in relation to CSVD and cognitive impairment, recent studies have increasingly emphasized multiparametric imaging models involving DTI-ALPS, EPVS, and other CSVD-related markers (11, 17). However, few studies have integrated these complementary diffusion-derived markers with conventional structural MRI markers to identify cognitive impairment in CSVD. In this study, we combined conventional structural MRI with 32-direction DTI to evaluate whether DTI-ALPS and WM-FW, individually and in combination, are associated with cognitive status in elderly patients with CSVD. Our primary objectives were to quantify the ALPS-Index and FW fraction, determine their relationships with established structural markers, including EPVS burden, Fazekas score, and lacunar infarcts, and assess whether their integration improves the identification of cognitive impairment beyond traditional lesion-based CSVD markers.

## Materials and methods

### Participants

This prospective study was approved on 17 January 2023 by the local ethics committee of Shanghai Punan Hospital of Pudong New Area (PN2022002), and informed consent was obtained from all participants in accordance with the Declaration of Helsinki. Between September 2024 and October 2025, we consecutively recruited 122 elderly individuals who presented for brain MRI at our Department of Neurology. Simultaneously, we recruited 60 healthy volunteers from the local community to serve as healthy controls (HC).

The inclusion criteria for the CSVD group were as follows: (1) age ≥ 50 years; (2) presence of at least one MRI marker of CSVD, including WMH, lacunar infarcts, or EPVS, according to STandards for ReportIng Vascular changes on nEuroimaging-2 (STRIVE-2) criteria (3); and (3) availability of a complete standardized cognitive evaluation. The inclusion criteria for the HC group were as follows: (1) age ≥ 50 years; (2) absence of any visible CSVD markers on MRI (Fazekas score = 0, no lacunes, and minimal EPVS); and (3) a Montreal Cognitive Assessment (MoCA) score ≥ 28.

Common exclusion criteria for all participants were as follows: (1) history of major territorial stroke, intracranial tumors, or non-vascular neurodegenerative diseases; (2) presence of metal implants or MRI contraindications; and (3) MRI scans of insufficient quality due to motion or susceptibility artifacts.

**Figure 1:**
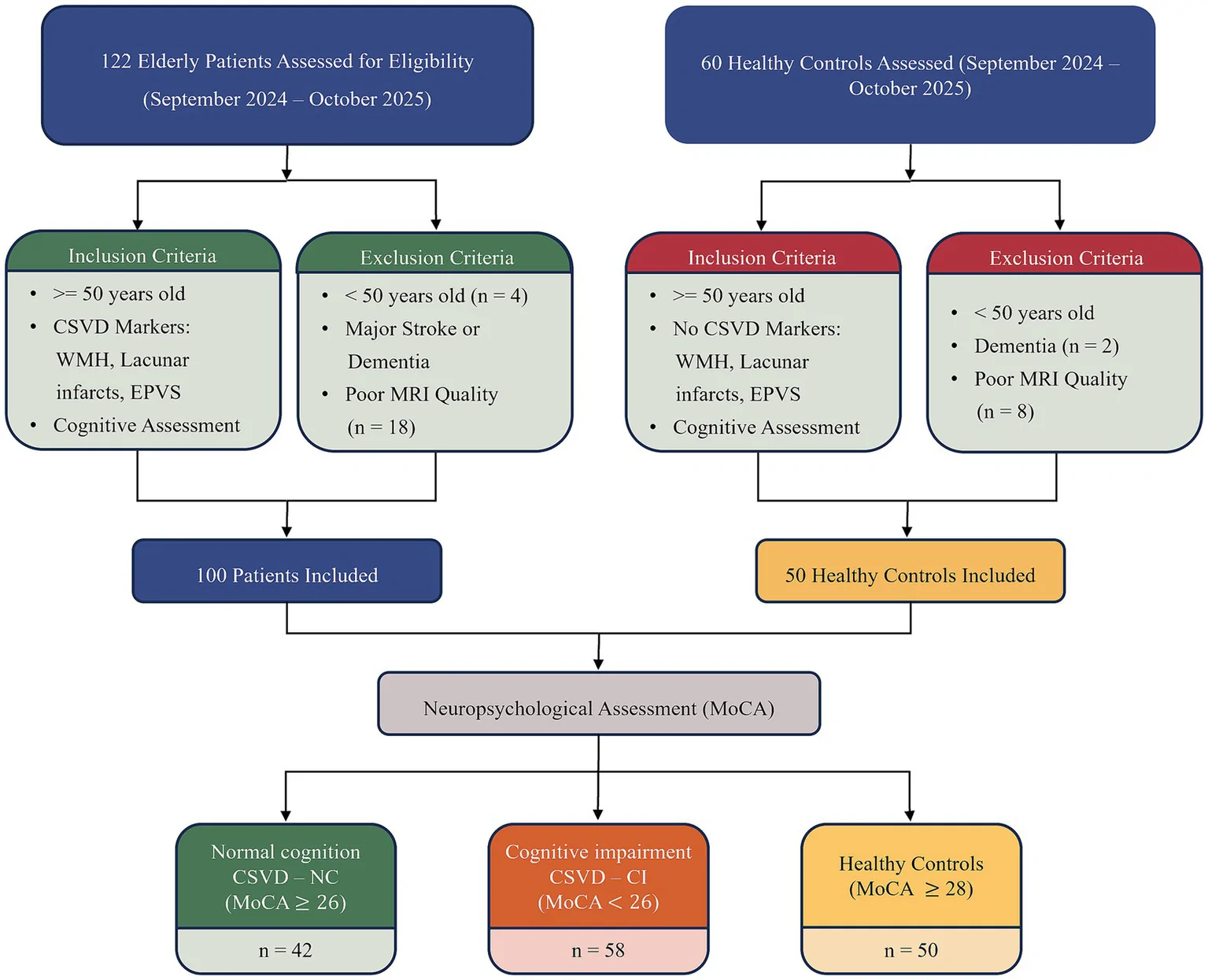
Flowchart of participant enrollment and grouping.

Following these criteria, from the initial patient pool, we excluded 4 participants under the age of 50, 8 who did not complete the full protocol, and 10 due to motion artifacts, leaving 100 patients with CSVD. From the control pool, 10 participants were excluded due to incidental findings or motion artifacts, resulting in a final cohort of 50 Healthy Controls (HC). The complete enrollment workflow is presented in Figure 1.

### Clinical and neuropsychological assessment

Two neurologists (6 years’ experience and 10 years’ experience, respectively) performed all clinical evaluations within 24 h of the MRI examination to ensure that the neuropsychological status reflected the participants’ current neuroimaging profile. We utilized the MoCA as the primary instrument for global cognitive screening because of its high sensitivity to the executive and visuospatial deficits characteristic of CSVD (18, 19). The assessment evaluated multiple cognitive domains, including visuospatial/executive functions, naming, attention, language, abstraction, delayed memory, and orientation (18). We applied a 30-point scoring scale and added one additional point for participants with 12 or fewer years of formal education to minimize educational bias (18). Age-adjusted or culture-specific normative cutoffs were not applied. Additional domain-specific neuropsychological tests were not available; therefore, cognitive grouping was based on MoCA screening rather than a formal neuropsychological diagnosis of vascular cognitive impairment. Participants were stratified based on adjusted MoCA scores into normal cognition (CSVD-NC, MoCA ≥ 26) and cognitive impairment (CSVD-CI, MoCA < 26) groups (18), as shown in Figure 1. Sleep status was assessed using the Epworth Sleepiness Scale (ESS). Demographic data and vascular risk profiles were also recorded. Hypertension was defined as systolic blood pressure ≥140 mmHg or diastolic blood pressure ≥90 mmHg, and diabetes mellitus was defined as fasting blood glucose ≥7.0 mmol/L or prior use of hypoglycemic agents (20, 21). Hypertension and diabetes mellitus were recorded because these variables were consistently available for all participants and are established vascular risk factors for CSVD. Other relevant vascular/metabolic factors, including hyperlipidemia, smoking status, and obesity, were not uniformly available for all participants and were therefore not included in the primary analysis.

### MRI acquisition

All examinations were performed on a 3.0-Tesla MRI system (uMR 870, United Imaging Healthcare, Shanghai, China) equipped with a 24-channel head–neck coil. The imaging protocol included 3D T1-weighted imaging, axial T2-weighted imaging, and axial T2-fluid-attenuated inversion recovery (FLAIR) imaging for structural evaluation of CSVD markers and anatomical guidance for region-of-interest placement. Diffusion imaging was then acquired using a 32-direction axial diffusion tensor imaging (DTI) sequence with two b0 images using sensitivity encoding (SENSE) acceleration to assess fluid-related microstructural alterations. The detailed imaging parameters are summarized in Table 1.

**Table 1:** MRI protocols.

Sequence	3D T1w GRE	T2w TSE	T2-FLAIR	DTI 32-dir
TR/TE/TI (ms)	8.0/3.1/750	6,225/141/−	8,000/106/2,425	4,809/88.4/−
Flip angle (degree)	9	90	90	90
Field of view (mm^2^)	256 × 256	230 × 200	230 × 200	256 × 256
Acquisition matrix	256 × 256	384 × 301	288 × 213	128 × 128
In-plane resolution (mm^2^)	1.0 × 1.0	0.60 × 0.66	0.80 × 0.94	2.0 × 2.0
Slice thickness (mm)	1.0	5.0	5.0	2.0
Slices	160	21	19	75
Echo train length	160	28	34	–
*b*-value (s/mm^2^)	–	–	–	0/1,000
Acceleration factor (Sense)	3	2	2	2
Scan time (min:s)	3:25	3:50	2:56	5:38

FLAIR: fluid attenuated inversion recovery; GRE: gradient echo sequence; TSE: turbo spin echo sequence; Sense: sensitivity encoding.

### CSVD marker assessment

The assessment of CSVD markers was conducted at an individual level by an experienced radiologist (WG, 10 years’ experience in neurology) who was blinded to cognitive grouping and diffusion-derived metrics. For the WMH, the Fazekas scale (ranging from 0 to 3) was applied to FLAIR images, following established guidelines (2). Cerebral infarcts were categorized as either territorial or lacunar, utilizing a scoring system based on the number of lacunar infarcts visible on FLAIR images (0 for none, 1 for ≤3, 2 for ≤10, and 3 for >10) (22). EPVS were rated separately in basal ganglia and centrum semiovale scores on T2-weighted images according to the Potter scale (23). For each region, EPVS status was graded as 0 (no EPVS), 1 (1–10 EPVS), 2 (11–20 EPVS), 3 (21–30 EPVS), and 4 (>40 EPVS) (24). The basal ganglia and centrum semiovale scores were then summed to generate a global EPVS burden score ranging from 0 to 8, following prior CSVD studies that used combined regional EPVS burden as an MRI-visible marker of CSVD (24). This summed score was used as the EPVS component in the conventional CSVD marker model.

### Advanced diffusion modeling and post-processing

**Figure 2:**
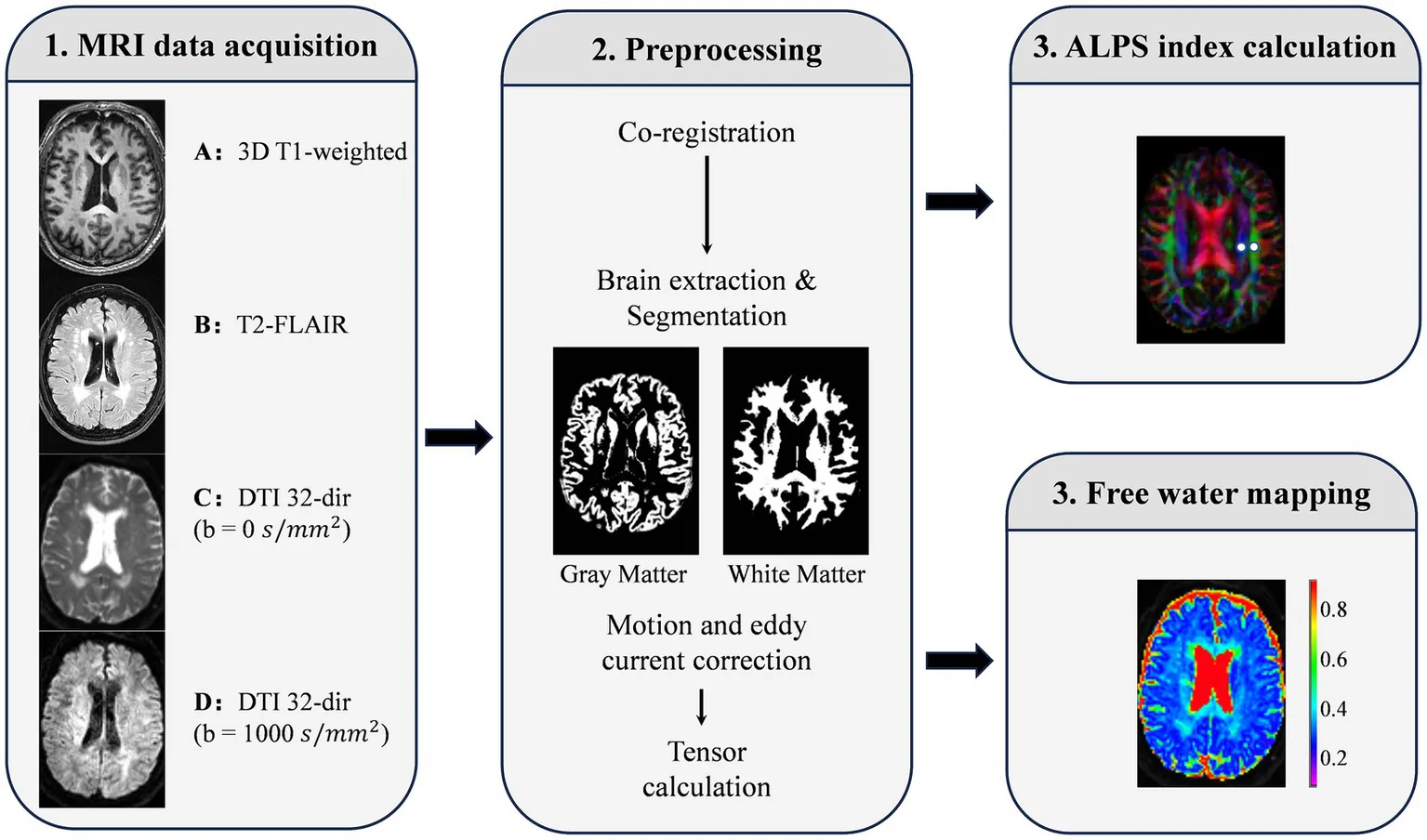
Multimodal MRI processing workflow and parameter estimation.

We preprocessed the raw diffusion data using the Functional Magnetic Resonance Imaging of the Brain (FMRIB) Software Library (FSL, version 6.0.7) for removal of non-brain tissues, motion correction, eddy current correction, and tensor calculation (25–27). The b-vectors were automatically rotated for tensor calculation during eddy-current and motion correction using the tool of eddy in FSL with the default setting. To assess the diffusion-derived surrogate marker, we calculated the ALPS-Index using bilateral ROIs placed at the level of the lateral ventricular body (Figure 2) (28). In each hemisphere, two spherical ROIs with a diameter of 5 mm were manually placed in the projection fiber region and association fiber region on color-coded fractional anisotropy maps in native diffusion space. Thus, four ROIs were used for each participant. ROI placement was performed by an experienced radiologist who was blinded to cognitive grouping and clinical information, and all ROIs were visually checked to avoid overt lesions, severe distortion, and CSF spaces. For each hemisphere, the unilateral ALPS-Index was calculated as the ratio of the mean x-axis diffusivity in the projection and association fiber ROIs to the mean y-axis diffusivity in the projection fiber ROI and z-axis diffusivity in the association fiber ROI (28). The final ALPS-Index was calculated as the average of the left and right unilateral ALPS-Index values.

To assess extracellular FW accumulation and interstitial edema burden, we applied the FW-DTI model (14, 29) using the MarkVCID FW kit (30). This single-shell regularized bi-tensor FW-DTI model estimates the isotropic FW fraction by partitioning the diffusion signal into an isotropic FW compartment and a hindered tissue compartment. The isotropic FW compartment was modeled with a fixed diffusivity of 3.0 × 10^−3^ mm^2^/s, the FW fraction was constrained between 0 and 1, and the tissue tensor was constrained to be positive definite. WM-FW, defined as the mean FW fraction within the whole-white-matter mask, was used as the quantitative FW-derived metric in this study. WM masks were generated from high-resolution 3D T1-weighted images using the Segment module in SPM12 implemented in MATLAB R2022b. The resulting WM segmentation outputs were binarized to generate masks covering the entire WM region. These masks were then co-registered to native diffusion space using the Coregister module in SPM12. WM-FW was calculated as the mean FW fraction within the co-registered WM mask. To address potential WMH-related or partial-volume effects, we performed an additional sensitivity analysis in which WM-FW was recalculated after excluding visible WMH voxels, periventricular voxels (±2 mm extension of WM mask adjacent to ventricles), or voxels adjacent to CSF (±2 mm extension of WM mask adjacent to CSF). The intraclass correlation coefficients of WM-FW values before and after exclusion were all greater than 0.95.

The overall MRI processing workflow and parameter estimation are illustrated in Figure 2.

### Statistical analysis

Statistical analyses were performed using SPSS (v27.0) and R software (v4.4.0). Continuous variables were tested for normality using the Shapiro–Wilk test. Normally distributed variables were expressed as mean ± standard deviation and compared among groups using ANCOVA with age, sex, education, hypertension, and diabetes as covariates, followed by Bonferroni-corrected *post hoc* comparisons. Non-normally distributed variables were expressed as the median (interquartile range). For group comparisons of non-normally distributed variables while adjusting for age, sex, education, hypertension, and diabetes, we used Quade’s non-parametric ANCOVA. Categorical variables were expressed as counts (percentages) and compared using the chi-square test or Fisher’s exact test, as appropriate.

Primary correlation analyses were performed across the overall CSVD cohort (*n* = 100). Additional exploratory subgroup correlation analyses were performed separately in cognitively normal CSVD participants and healthy controls to assess whether associations between MoCA score and diffusion-derived metrics were detectable in participants without MoCA-defined cognitive impairment. MoCA and ESS scores were analyzed as continuous outcomes. Pearson correlation and partial correlation analyses (adjusting for age, sex, education, hypertension, and diabetes) were performed to investigate the associations of the ALPS-Index and WM-FW with MoCA and ESS. Multivariate logistic regression models were then constructed to identify factors significantly associated with cognitive impairment, including age, sex, education, hypertension, and diabetes as covariates. Model discrimination was summarized by receiver operating characteristic (ROC) curves with area under the curve (AUC) and 95% CIs derived from 1,000 bootstrap resamples; AUCs were interpreted as excellent (>0.9), good (0.8–0.9), moderate (0.7–0.8), fair (0.6–0.7), and poor (< 0.6). Pairwise comparisons of AUCs between traditional CSVD burden scores and combined “glymphatic-edema signature” (ALPS-Index and WM-FW) were performed using the DeLong test. All tests were two-sided with a significance level set at *p* < 0.05.

## Results

### Participant characteristics

The study population comprised 100 patients with CSVD and 50 HCs. As summarized in Table 2, the CSVD-CI group was significantly older than the CSVD-NC group (*p* < 0.001), whereas sex distribution, educational level, and hypertension and diabetes were comparable. In addition, the CSVD-CI group showed lower MoCA scores and higher ESS scores than the CSVD-NC group. The burden of conventional CSVD markers, especially WMH and EPVS, was also greater in the CSVD-CI group.

**Table 2:** The demographic, clinical, and neuroimaging characteristics of all participants.

CSVD patients (*n* = 100) and HC (*n* = 50)				
Variables	CSVD-CI (*n* = 58)	CSVD-NC (*n* = 42)	HC (*n* = 50)	CSVD-CI vs. CSVD-NC *p*-value
Demographics				
Age (years)	68.03 ± 8.09	60.14 ± 8.90	64.32 ± 7.90	<0.001
Gender (male/female)	24/34	18/24	20/30	0.88
Education (years)	9.30 ± 2.13	9.93 ± 2.77	10.22 ± 2.51	0.22
Hypertension (with/without)	22/36	16/26	17/33	0.99
Diabetes (with/without)	14/44	10/32	10/40	0.97
Clinical scores				
MoCA	23.20 ± 1.00	29.33 ± 0.57	29.53 ± 0.43	<0.001
ESS	12.17 ± 2.62	10.05 ± 2.85	8.21 ± 2.05	<0.001
Fazekas score (0/1/2/3)	0/32/20/6	8/22/10/2	NA	0.003
Lacunar infarct (0/1/2/3)	36/16/4/2	32/10/0/0	NA	0.17
EPVS (0/1/2/3/4/5/6/7/8)	0/0/4/9/10/13/11/7/4	0/0/8/12/10/7/5/0/0	NA	0.023

CI: cognitive impairment; CSVD: cerebral small vessel disease; EPVS, enlarged perivascular space; ESS: Epworth sleepiness scale; HC: healthy control; MoCA: Montreal Cognitive Assessment; NA: not available.

### Functional diffusion metrics across groups

**Figure 3:**
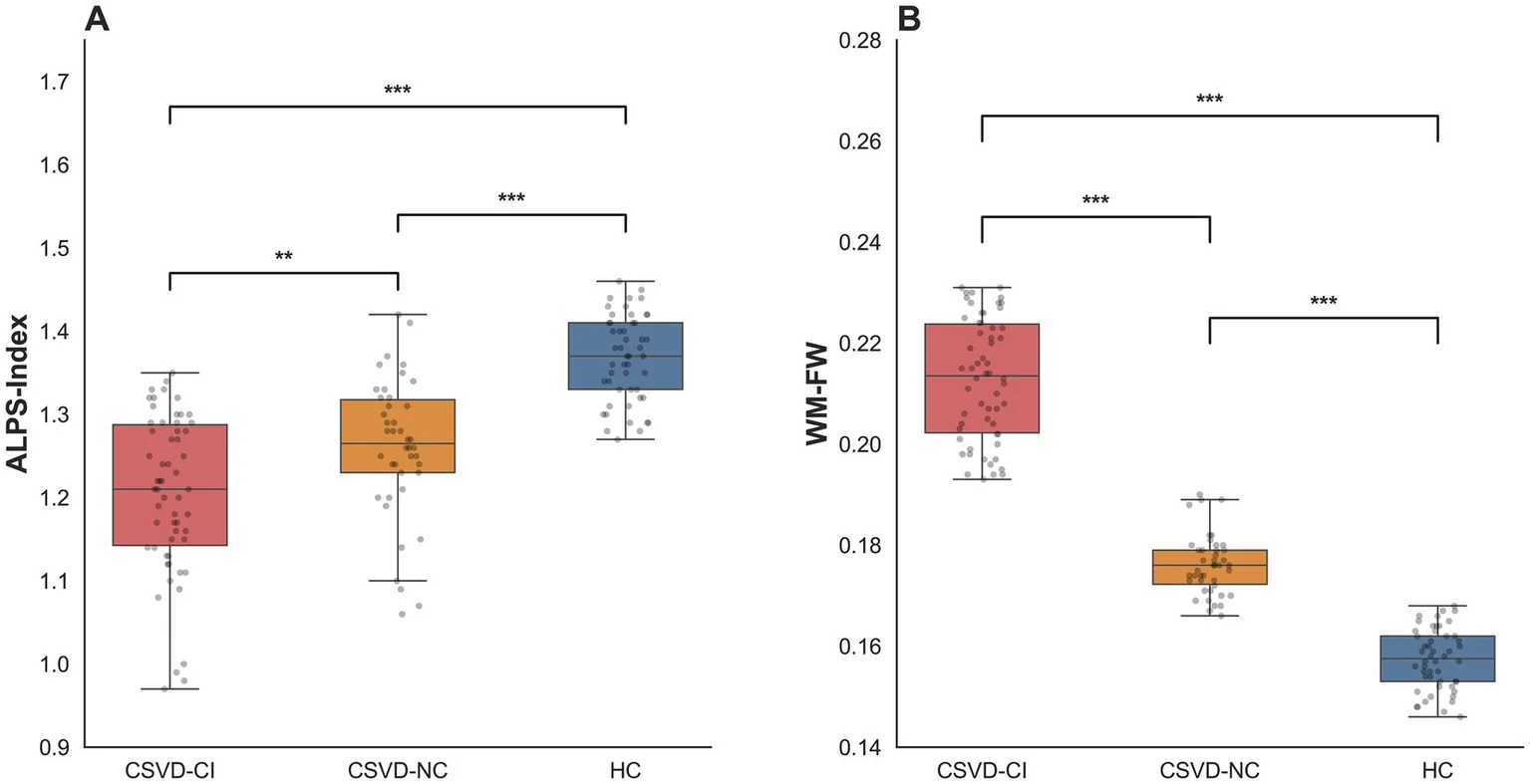
Comparisons of ALPS-Index and WM-FW across groups. Boxplots illustrate the distributions of ALPS-Index **(A)** and WM-FW **(B)** in the CSVD-CI (*n* = 58), CSVD-NC (*n* = 42), and HC (*n* = 50) groups. Pairwise comparisons showed significant differences among all three groups for both metrics.

Quantitative analysis of the functional diffusion metrics revealed significant group differences, Figure 3. The ALPS-Index showed a stepwise decrease from HC to CSVD-NC and CSVD-CI, with mean values of 1.368 ± 0.052, 1.260 ± 0.085, and 1.204 ± 0.095, respectively, Figure 3A. In contrast, WM-FW increased progressively from HC to CSVD-NC and CSVD-CI, with mean values of 0.157 ± 0.006, 0.176 ± 0.006, and 0.213 ± 0.012, respectively, Figure 3B. ANCOVA and post-hoc pairwise comparisons showed significant differences among all three groups for both metrics (all *p* < 0.001).

### Correlations between functional diffusion metrics and cognition and sleep status

**Figure 4:**
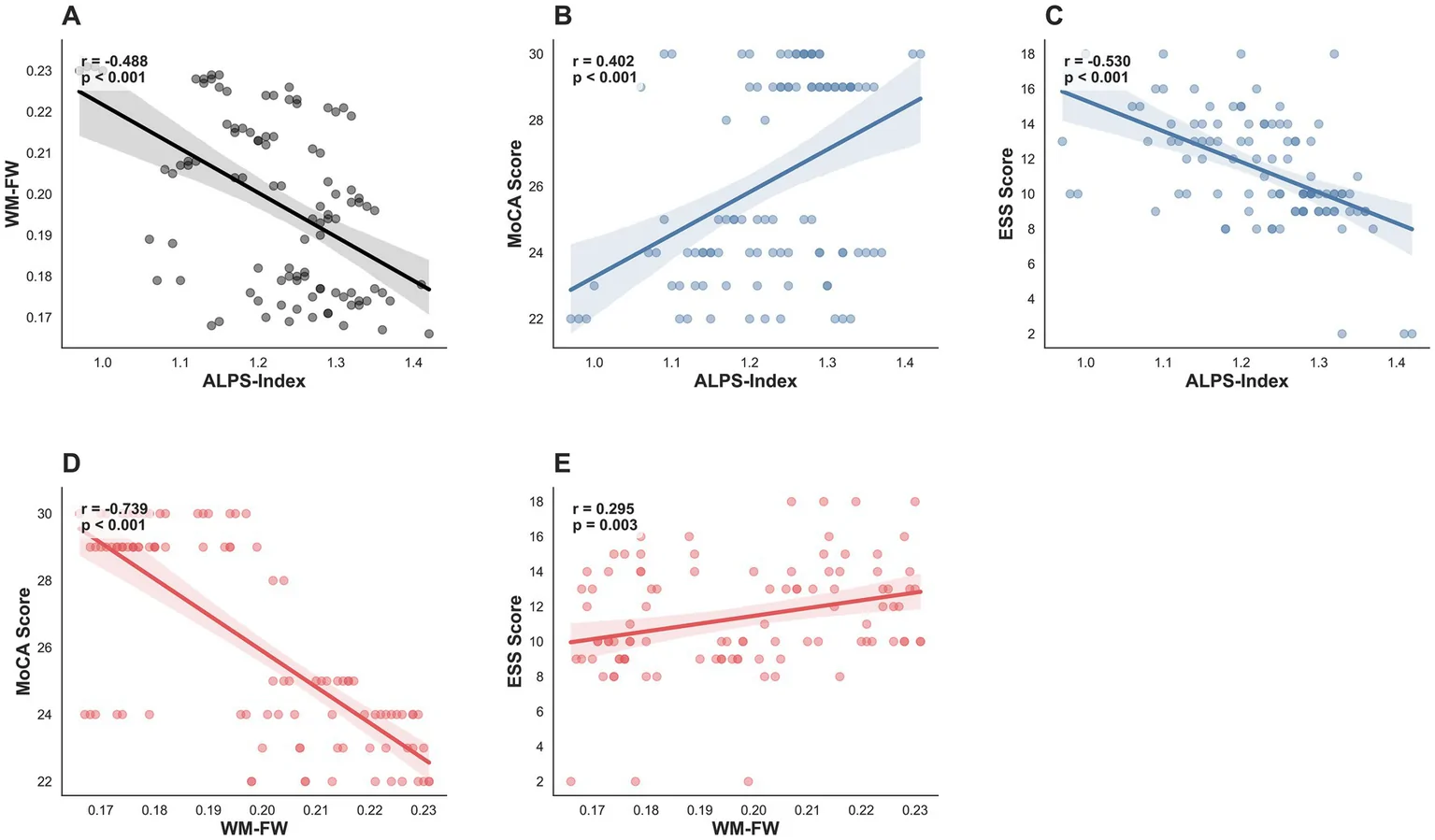
Pearson correlation analysis of functional and clinical metrics in the CSVD cohort (*n* = 100). Scatter plots show the associations between ALPS-Index and WM-FW **(A)**, ALPS-Index and global cognition assessed by MoCA **(B)**, ALPS-Index and daytime sleepiness assessed by ESS **(C)**, WM-FW and global cognition assessed by MoCA **(D)**, and WM-FW and daytime sleepiness assessed by ESS **(E)**.

In the overall CSVD cohort (*n* = 100), both Pearson correlation and partial correlation analyses demonstrated significant associations among ALPS-Index, WM-FW, cognitive performance, and daytime sleepiness (Figure 4). Lower ALPS-Index was significantly associated with higher WM-FW fraction (*r* = −0.488, *p* < 0.001; partial *r* = −0.361, *p* = 0.001; Figure 4A), lower MoCA score (*r* = 0.402, *p* < 0.001; partial *r* = 0.263, *p* = 0.005; Figure 4B), and higher ESS score (*r* = −0.53, *p* < 0.001; partial *r* = −0.661, *p* < 0.001; Figure 4C). In contrast, higher WM-FW was negatively correlated with lower MoCA score (*r* = −0.739, *p* < 0.001; partial *r* = −0.620, *p* < 0.001; Figure 4D) and higher ESS score (*r* = 0.295, *p* = 0.003; partial *r* = 0.213, *p* = 0.025; Figure 4E). In additional subgroup analyses performed separately in cognitively normal CSVD participants and healthy controls, no significant correlations were observed between MoCA score and ALPS-Index or WM-FW after Bonferroni correction, with all Bonferroni-corrected *p* values > 0.05.

### Discriminative performance for MoCA-defined cognitive impairment

**Figure 5:**
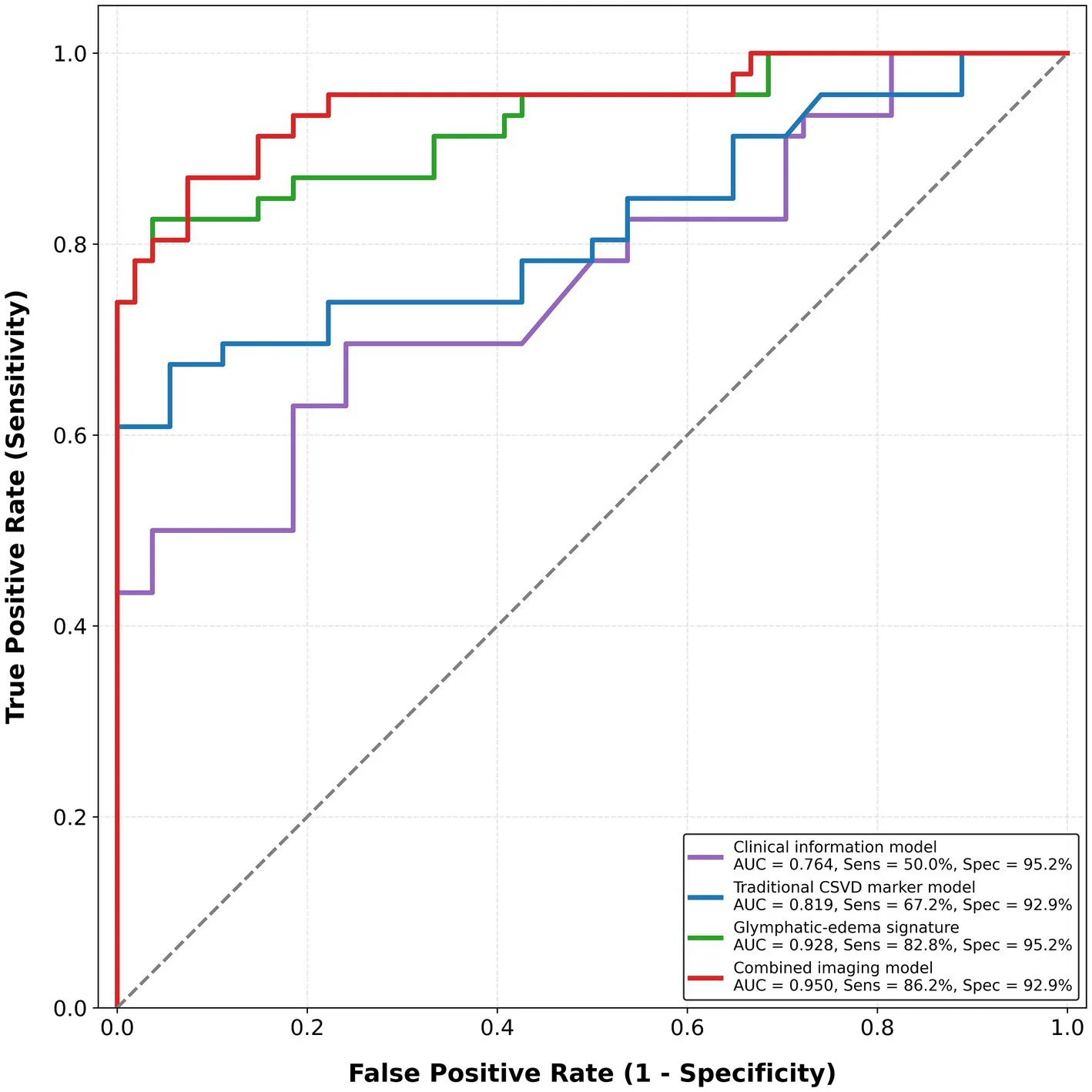
Discriminative performance for identifying MoCA-defined cognitive impairment in CSVD patients. ROC curves illustrate the discriminative performance of the clinical information model, traditional CSVD markers model, glymphatic-edema signature incorporating both ALPS-Index and WM-FW, and combined imaging model integrating both functional and structural markers. The combined imaging model achieved the highest AUC.

The results of the multivariate logistic regression analysis, detailed in Table 3, confirmed that both traditional CSVD markers (Fazekas score and lacunar infarcts) and advanced functional diffusion metrics (ALPS-Index and WM-FW) serve as significant predictors of cognitive status. The comparative diagnostic utility of these metrics was further evaluated via multivariate ROC analysis, as shown in Figure 5. The clinical information model (age, sex, education, hypertension, and diabetes) provided the baseline with an AUC of 0.764 (95% CI: 0.669–0.859). When including age, sex, education, hypertension, and diabetes as covariates, the traditional CSVD marker model shows good discrimination with an AUC of 0.819 (95% CI: 0.731–0.908), and the integration of the glymphatic-edema signature—comprising both the ALPS-Index and WM-FW—yielded a significantly higher AUC of 0.928 (95% CI: 0.875–0.981, *p* < 0.001). Furthermore, the combined imaging model incorporating traditional CSVD markers with ALPS-Index and WM-FW achieved the highest discriminative performance, with an AUC of 0.950 (95% CI: 0.906–0.993). Under five-fold cross validation, the AUCs of clinical information, traditional CSVD marker, diffusion-derived, and combined imaging models became 0.698 ± 0.091, 0.736 ± 0.104, 0.845 ± 0.113, and 0.889 ± 0.096, respectively.

**Table 3:** Multivariate logistic regression analyses of imaging variables associated with cognition assessment in CSVD patients.

Variables	***β*** coefficient (95% CI)	OR (95% CI)	Bonferroni corrected *p*-value
Fazekas score (0/1/2/3)	0.96 (0.10, 1.88)	2.60 (1.11, 6.53)	0.043
Lacunar infarct (0/1/2/3)	1.76 (0.58, 3.09)	5.79 (1.79, 21.94)	0.012
EPVS (0/1/2/3/4/5/6/7/8)	0.35 (−0.09, 0.80)	1.41 (0.91, 2.23)	>0.1
ALPS-Index	−2.01 (−4.14, −0.21)	0.13 (0.016, 0.81)	0.008
WM-FW	3.23 (1.27, 5.66)	25.22 (8.57, 88.20)	<0.001

ALPS: diffusion analysis along the perivascular space; CSVD: cerebral small vessel disease; EPVS, enlarged perivascular space; WM: white matter; FW: free water.

## Discussion

This study showed that reduced ALPS-Index and elevated WM-FW are both associated with cognitive impairment in elderly patients with CSVD. Compared with cognitively normal patients and HCs, cognitively impaired patients exhibited lower ALPS-Index and higher WM-FW, and both metrics were significantly related to cognitive performance and daytime sleepiness. In addition, the integration of ALPS-Index and WM-FW improved the identification of cognitive impairment beyond conventional CSVD imaging markers alone. These findings support the view that altered perivascular diffusion and extracellular FW accumulation represent complementary imaging features of CSVD-related cognitive vulnerability.

The reduction in ALPS-Index across cognitive stages is consistent with previous studies indicating that a lower ALPS-Index is associated with CSVD burden and cognitive decline (9, 10, 31). Recent studies have further linked reduced ALPS-Index to greater perivascular space burden, hypertension-related CSVD changes, and different cognitive stages of CSVD (11, 12). The ALPS method provides a diffusion-based surrogate of perivascular fluid transport rather than a direct measurement of glymphatic clearance. In our cohort, ALPS-Index decreased stepwise from healthy controls to cognitively normal CSVD and cognitively impaired CSVD, and lower ALPS values were associated with lower MoCA scores and higher ESS scores. The significant between-group difference in ALPS-Index between HCs and CSVD-NC participants suggests that altered perivascular diffusion may already be detectable in cognitively preserved CSVD. Furthermore, the association between ALPS-Index and EPVS burden supports the interpretation that visible perivascular space enlargement may be related to altered perivascular diffusion rather than representing a purely static anatomical change (24, 32).

WM-FW provided an additional and, according to our data, stronger signal related to cognitive status. WM-FW reflects extracellular FW accumulation and has been associated with blood–brain barrier dysfunction, interstitial edema, and cognitive impairment in cerebrovascular disease (14, 16, 33). Recent longitudinal evidence in CSVD further suggests that WM-FW changes are related to cerebral blood flow and white matter fiber alterations (34). In the present study, WM-FW increased progressively from healthy controls to cognitively normal CSVD and cognitively impaired CSVD, and higher WM-FW was significantly associated with lower MoCA scores and higher ESS scores. This result is consistent with recent evidence suggesting that free water may act as an intermediate imaging marker linking perivascular diffusion to cognitive decline (35). The significant inverse relationship between ALPS-Index and WM-FW in our cohort further supports the possibility that altered perivascular diffusion and extracellular FW accumulation are coupled processes in CSVD (6–8, 36). Compared with conventional lesion counting, WM-FW may therefore provide greater sensitivity to tissue changes occurring in normal-appearing WM before overt structural progression becomes evident (15, 16, 33, 37). Despite the significant between-group differences in ALPS-Index and WM-FW between HCs and CSVD-NC participants, within-group correlations between MoCA score and either diffusion-derived metric were not significant in either subgroup after Bonferroni correction, possibly because of the restricted MoCA range and potential ceiling effect.

An important finding of this study is that the glymphatic-edema signature, defined by the integration of ALPS-Index and WM-FW, improved discriminative performance relative to conventional CSVD markers. Although previous studies have investigated individual diffusion-derived metrics in CSVD, our study emphasizes the incremental value of integrating two complementary diffusion-derived markers—perivascular diffusion and extracellular FW burden—with conventional structural MRI markers. This result suggests that lesion-based markers and diffusion-derived surrogate markers capture partly distinct aspects of disease burden. Conventional MRI markers such as WMH, lacunar infarcts, and EPVS describe accumulated structural injury, whereas ALPS-Index and WM-FW may reflect altered perivascular diffusion and extracellular FW burden (6, 9, 14, 36). Their combination therefore provides a more informative framework for identifying MoCA-defined cognitive impairment in CSVD than either category alone. Rather than replacing traditional CSVD markers, these diffusion-derived metrics may serve as complementary biomarkers that enhance imaging-based characterization of cognitive vulnerability.

The associations with ESS further suggest that altered perivascular diffusion and extracellular FW burden may extend beyond cognition to sleep-related dysfunction in older adults with CSVD. Experimental and clinical studies have shown that glymphatic activity is closely linked to sleep, and impaired glymphatic function has been associated with poorer sleep quality and cognitive decline in aging populations (38, 39). In our cohort, ALPS-Index was negatively associated with ESS, indicating that lower perivascular diffusion was related to greater daytime sleepiness. In contrast, WM-FW was positively associated with ESS, suggesting that greater extracellular FW accumulation was also associated with daytime sleepiness. Although ESS does not directly measure nocturnal sleep architecture, these findings support the possibility that sleep-related symptoms may be associated with altered perivascular diffusion and extracellular FW accumulation in CSVD (38, 39).

Several limitations should be acknowledged. First, the cross-sectional design precludes causal inference, and longitudinal studies are needed to determine whether ALPS-Index reduction and WM-FW elevation precede cognitive decline or simply accompany it. Second, FW estimation was based on a single-shell regularized bi-tensor model. Although this is an established approach for FW imaging, it is less robust than multi-shell acquisition and may be more susceptible to model instability, bias, and partial-volume effects; therefore, the WM-FW results should be interpreted cautiously (40). Third, the susceptibility-distortion correction and Gibbs ringing correction were not performed during DTI preprocessing. The ALPS approach depends on the accuracy of ROI placement defined by a single trained radiologist and therefore remains susceptible to operator-dependent variation and structural distortion in diseased brains (9, 28). In addition, because the two ROIs were placed near the body of the lateral ventricle, directional diffusivity estimates may be affected by CSF-related partial-volume contamination. Because these ROIs are anatomically closer to the centrum semiovale, the use of a summed EPVS score without separate BG-EPVS and CSO-EPVS analyses is another limitation. Fourth, although the present sample size was adequate for group comparisons and ROC analysis, external validation in larger and multicenter cohorts is still required. In addition, vascular/metabolic covariates beyond hypertension and diabetes mellitus, such as hyperlipidemia, smoking status, and obesity, were not uniformly available and therefore were not included in the present analyses, which may have introduced residual confounding. Finally, while our findings support the value of combining ALPS-Index and WM-FW, the biological interpretation of these metrics remains indirect, and future work integrating multimodal imaging, longitudinal follow-up, and possibly biochemical or vascular markers will be important to clarify their mechanistic significance.

In summary, this study suggests that reduced ALPS-Index and increased WM-FW represent two complementary diffusion-derived imaging manifestations of CSVD-related cognitive impairment. By integrating DTI-ALPS and WM-FW with traditional MRI markers, we obtained improved identification of MoCA-defined cognitive impairment in elderly patients with CSVD. These findings support the potential of diffusion-derived imaging biomarkers to enrich traditional structural assessment and to provide a more biologically informed evaluation of cognitive vulnerability in CSVD.

## Data Availability

The data analyzed in this study are subject to privacy and institutional restrictions. Raw de-identified imaging data and ROI masks cannot be publicly shared because of participant privacy and institutional data-governance requirements. Derived quantitative metrics and analysis code are available from the corresponding author upon reasonable request, subject to institutional approval and a data-use agreement.
