# Correction: Perivascular diffusion and white matter free water improve identification of cognitive impairment in cerebral small vessel disease

**DOI:** 10.3389/fneur.2026.1973478

**Published:** 2026-09-15

**Authors:** Chunhong Yang, Sunmei Cai, Bin Wang, Jie Zhou, Dongmei Wu, Muzi Li, Wenlu Geng, Lianshuang Wu, Kaiyue Peng, Yi Yang, Wenquan Gu

**Affiliations:** 1 Department of Radiology, Punan Branch of Renji Hospital, Shanghai Jiaotong University School of Medicine (Punan Hospital in Pudong New District, Shanghai), Shanghai, China; 2 Shanghai Key Laboratory of Magnetic Resonance, School of Physics, Institute of Magnetic Resonance and Molecular Imaging in Medicine, East China Normal University, Shanghai, China; 3 School of Engineering and Applied Science, University of Pennsylvania, Philadelphia, PA, United States; 4 Department of Neurology, Punan Branch of Renji Hospital, Shanghai Jiaotong University School of Medicine (Punan Hospital in Pudong New District, Shanghai), Shanghai, China; 5 Department of Nuclear Medicine, The First Affiliated Hospital of Soochow University, Suzhou, China

**Keywords:** cerebral small vessel disease, cognitive impairment, diffusion tensor imaging, free water, glymphatic system, neuroimaging biomarkers

An incorrect grant number was provided for the Health Scientific Research Project of the Pudong New Area Health Commission, General Project. The grant number was erroneously given as “20251021112237092”. The correct grant number is “PW2025A-16”.

The correct Funding statement is:

